# The Ability of Polyuria in Prediction of Weaning Outcome in Critically Ill Mechanically Ventilated Patients

**Published:** 2019-01

**Authors:** Masoud Aliyali, Ali Sharifpour, Siavash Abedi, Fatemeh Spahbodi, Narges Namarian, Adel Zarea, Ahad Alizadeh

**Affiliations:** 1 Internal Medicine Department, Pulmonary and Critical Care Division, Mazandaran University of Medical Sciences, Sari, Iran; 2 Internal Medicine Department, Nephrology Division, Mazandaran University of Medical Sciences, Sari, Iran; 3 Internal Medicine Department, Mazandaran University of Medical Sciences, Sari, Iran

**Keywords:** Fluid balance, Polyuria, Weaning, Mechanical ventilation

## Abstract

**Background:**

Fluid balance and oliguria influence outcome in critically ill patients. Although, osmotic dieresis with hypernatraemia is a predictor of mortality in critically ill patients, the purpose of this study was to demonstrate the effect of polyuria as an independent predictor on weaning outcome in mechanically ventilated patients.

**Materials and Methods:**

This retrospective, single center, cohort study was carried out at Imam Teaching Hospital Intensive Care Unit (ICU) on 263 adult mechanically ventilated patients. We collected data of these patients during the mean seven consecutive days before weaning from mechanical ventilator. Patients with polyuria (sustained urine output greater than 3000 ml/day) were compared with patients without polyuria. The primary endpoint was successful weaning and the secondary endpoints were the mechanical ventilation duration, post weaning length of ICU stay, post weaning length of hospitalization and rate of mortality.

**Results:**

In 93 patients with polyuria, the mean age was 45.14±19.47 years in comparison of 170 patients without polyuria with mean age of 52.9±21.37 years (P=0.004). Fluid intake, urine output and temperature were significantly higher in patients with polyuria, but there were no statistical differences in systolic and diastolic blood pressure, serum electrolytes, urea and creatinine. No significant differences were found in primary and secondary endpoints including successful weaning, post weaning length of ICU stay, post weaning hospital duration and mortality, except for duration of mechanical ventilation (P=0.014). The area under the ROC curve for variables showed only seven days mean creatinine level before weaning which may act as a predictor of successful weaning (ROCAUC=0.67, 95% CI 0.61–0.73, P=0.0002). Serum creatinine level of 0.8 provided best overall combination of sensitivity and specificity for successful weaning (sensitivity 72.22%, 95% CI 54.8–85.8; specificity 61.19%, 95% CI 54.1–68.0).

**Conclusion:**

Polyuria cannot predict weaning outcome but maybe considered as a predictor of longer duration of mechanical ventilation and is probably associated with a subclinical renal dysfunction.

## INTRODUCTION

As shown in previous study, fluid balance significantly influences outcome in critically ill patients. Positive cumulative fluid balance can influence clinical outcome of these patients during and after discharge from the Intensive Care Unit (ICU) and is probably a predictor of the severity of the underlying disease and failure of cardiovascular and renal system to eliminate excess fluid, especially in older ages (1,2). On the other hand, negative fluid balance is an independent risk factor for lower mortality and reduction in ICU stay and ventilator requirement especially in critically ill patients with acute kidney injury (3). Negative fluid balance was also associated with reduction mortality in acute care surgery patients (4). In addition of fluid balance, oliguria is associated with higher mortality in critically ill patients, especially in patients with acute kidney injury (5). On other hand, osmotic urea dieresis with consequent hypernatraemia is a predictor of mortality in critically ill patients (6). Although many etiologies can be identified in patients with polyuria, with two principal causes including aqueous polyuria and osmotic polyuria (7), we hypothesized patients with polyuria have probably more ability in fluid excretion and consequently more cardiovascular fitness.

The aim of this study was to evaluate the impact of polyuria on weaning outcome in mechanically ventilated patients as an independent predictor.

## MATERIALS AND METHODS

This retrospective, single center, cohort study was conducted in Imam Teaching Hospital ICU on adult mechanically ventilated patients from Jan. 2013 to Dec. 2015. The study protocol was approved by local ethics committee.

Subjects were included if they were older than 18 years of age and mechanically ventilated for more than 72 hours. Patients meeting the following criteria were excluded: renal dysfunction (Creatinine Cr>2 mg/dl), congestive heart failure, gastrointestinal bleeding, hemodynamic instability needing fluids and vasoactive agents, temperature more than 38.3°C, hypokalemia lower than 3.5 mmol/l, hypercalcemia more than 1.29 mmol/l, loop diuretics use, hyperglycemia more than 10 mmol/l, and administration of drugs associated with nephrogenic diabetes insipidus (amphotericin B, dexamethasone, rifampin, triamterene-hydrochlorothiazide).

We reviewed medical records of over 900 patients admitted to ICU during the study period and finally 263 patients were enrolled. Patients’ data obtained from medical and chart records consisted of demographics, ICU admission diagnosis, fluid intake, urine output, systolic and diastolic blood pressures, temperature, urea, creatinine, and serum electrolytes during the mean seven consecutive days before weaning from mechanical ventilator.

Patients were dichotomized into two groups as patients with sustained polyuria before first attempt for weaning and patients without polyuria. Polyuria was defined as urine output of greater than 3000 ml/day (8).

The primary outcome was successful weaning. The secondary endpoints were the length of mechanical ventilation, post weaning length of ICU stay, post weaning length of hospitalization and rate of mortality.

The data were presented as the mean±SD unless otherwise indicated. Continuous variable were compared using the Mann-Whitney U test and Student t test. Categorical variables were analyzed using X2 test and Fisher exact test. P<0.05 was considered to be statistically significant. Receiver-Operator Characteristic (ROC) curve analysis was used to assess the ability of variables to predict successful weaning from mechanical ventilator.

## RESULTS

Of the 263 enrolled patients, we found 93 patients with polyuria in comparison with 170 subjects without polyuria.

Demographic and pre-existing comorbid conditions are mentioned in table 1. There was a statistically significant difference in age (P=0.004) among two groups but other demographic and comorbidity were similar.

**Table 1.** Demographic and underling comorbidity among mechanically ventilated patients with and without polyuria

**Variable**	**With polyuria (N=93) (N %)**	**Without polyuria (N=170) (N %)**	**P value**
**Age, y (SD)**	45.14 (19.47)	52.9 (21.37)	0.004
**Sex**			
Male	71(76.3)	117(68.8)	0.197
Female	22 (23.7)	53(31.2)	
**Underlying comorbidity**			
CVA	0	9	0.018
IHD	4	21	0.062
Hypertension	22	57	0.033
COPD	4	12	0.371
Asthma	1	4	0.42
Diabetes mellitus	13	28	0.594
Hypothyroidism	1	2	0.714
Malignancy	6	17	0.213

As shown in table 2, fluid intake, urine output and temperature were significantly higher in patients with polyuria, but there were no statistical differences in systolic and diastolic blood pressure, serum electrolytes, urea and creatinine.

**Table 2.** Intake and output, hemodynamic and laboratory parameters between two groups

**Variable**	**With polyuria N=93**	**Without polyuria N=170**	**P value**
**Fluid intake**	3774.76	3316.65	<0.001
**Urine output**	3650.97	2178.07	<0.001
**Systolic blood pressure**	12.50	12.57	0.658
**Diastolic blood pressure**	7.43	7.36	0.578
**Creatinine**	0.84	0.86	0.526
**Urea**	32.39	34.15	0.359
**Temperature**	37.18	37.11	0.004
**Sodium**	138.6	138.5	0.927
**Potassium**	4.0	3.9	0.087

As demonstrated in table 3, no significant differences were found in primary and secondary endpoints including successful weaning, post weaning length of ICU stay, post weaning length of hospital stay and mortality, except for length of mechanical ventilation (P=0.014).

**Table 3.** Results of primary and secondary outcomes

**Variable**	**With polyuria N=93**	**Without polyuria N=170**	**P value**
**Successful weaning**	73(78.5%)	137(79.4%)	0.861
**Length of mechanical ventilation**	9.13	6.61	0.014
**Length of post weaning ICU stay**	6.08	4.92	0.169
**Length of post weaning hospitalization**	5.72	5.18	0.68
**Mortality**	7	17	0.33

**Figure 1.**
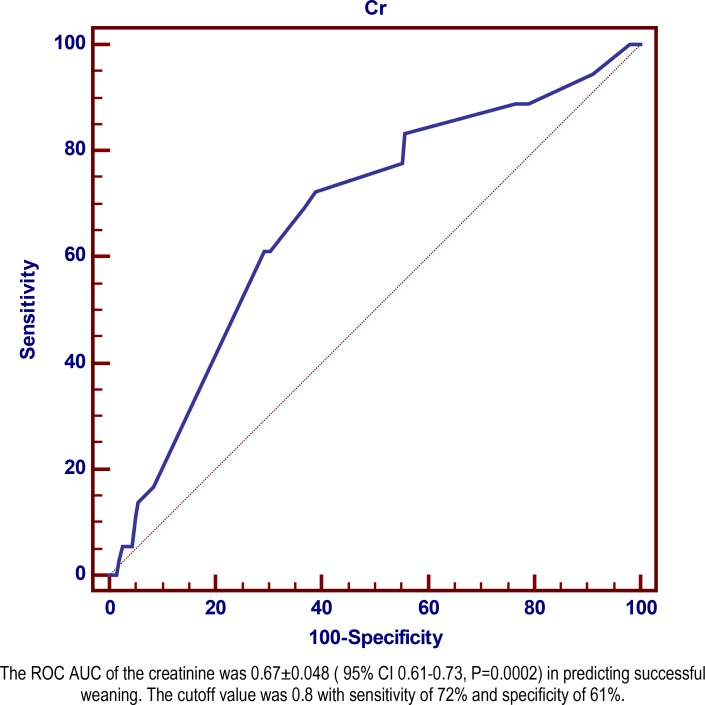
Receiver operating characteristic curve of serum creatinine as a predictor of successful weaning.

The area under the ROC curve for variables including creatinine, fluid balance, variation of output [(output − intake/intake) ×100], sodium and potassium showed only the mean seven days creatinine level before weaning may act as a predictor of successful weaning. However, the ROCAUC for creatinine to predict successful weaning (Figure 1) showed a statistically significant but poor predictive ability (ROCAUC=0.67, 95% CI 0.61–0.73, P=0.0002). Serum creatinine level of 0.8 provided best overall combination of sensitivity and specificity for successful weaning (sensitivity 72.22%, 95% CI 54.8–85.8; specificity 61.19%, 95% CI 54.1–68.0).

## DISCUSSION

Although we initially hypothesized patients with polyuria have probably more ability in fluid excretion and consequently more cardiovascular fitness, as shown in this study polyuria did not influence weaning success and these patients had longer mechanical ventilation duration. Thus, polyuria before weaning was not a positive predictor of successful weaning.

We propose several hypotheses for explaining of our study results. First, higher amount of intravascular fluid administration probably puts these patients in a higher steady state of total body water and this causes higher total lung water and probably pulmonary interstitial edema and longer need for mechanical ventilation. Of course, distribution of total body water depends on a lot of factors including osmolality, and relative sodium content; thus, amount of pulmonary interstitial edema cannot be simply predictable (9).

On the other hand, when fluid balance was compared in the two groups with and without polyuria, it was realized that patients without polyuria often had higher positive fluid balance with higher likelihood of pulmonary interstitial and probably other organs edema, so higher level of steady state of total body fluid and pulmonary interstitial edema could not merely explain longer duration of mechanical ventilation in patients with polyuria.

We should emphasize that total body water and pulmonary water were not measured in this study, so the contribution of this mechanism in longer duration of mechanical ventilation could not be analyzed precisely.

Another reason for unimproved outcome in polyuric patients was probably the inability of kidneys in proper urine concentration and this might represent somewhat a subclinical renal dysfunction with renal water loss. Of course, to complete this conclusion, it must be considered the presence of osmotic urea diuresis and also calculated electrolyte free water clearance (EFWC) to identify ongoing renal loss of free water and classic free water clearance (FWC) for assessing water retention by kidney (6).

Although, we did not demonstrate a clinically significant kidney injury in our study, data from other studies show that pulmonary complications are common and serious in patients with acute kidney injury with higher mortality and morbidity due to pulmonary edema, and respiratory failure requiring mechanical ventilation, prolonged mechanical ventilation, and prolonged weaning (10,11).

In spite of polyuric states, alterations of water and sodium metabolism and also acid-base balance consequently influence the outcome (7,12). In this study we could not find any difference and alterations in the hemodynamic state, serum urea, creatinine, sodium, and potassium in patients with polyuria in comparison with patients without polyuria. Hypernatremia with related polyuria is an independent predictor of higher mortality in critically ill patients (6,13), but as shown in our study polyuria without dysnatremia also can influence some outcomes including length of mechanical ventilation and somewhat post weaning length of ICU stay.

As mentioned, there might be a subclinical renal dysfunction in patients with polyuria. The possibility of this hypothesis rises when according to ROC curve analysis among studied variables including creatinine, fluid balance, output variation, sodium and potassium, only serum creatinine had sensitivity and specificity to predict successful weaning from mechanical ventilation. Thus, maximum renal function may have an important role in successful weaning from mechanical ventilation. However, as shown subclinical renal dysfunction did not influence final outcomes including post weaning length of hospital stay and mortality.

As demonstrated in our study, patients with polyuria had lower positive fluid balance, but this was not associated with positive effect on the outcome. Thus, it seems as long as a positive fluid balance is maintained the prognosis could not improve and probably only a negative fluid balance might lower mortality and improve weaning outcome (14,15).

Of course, as demonstrated in our study, patients with polyuria were younger than patients without polyuria and larger amounts of intravenous fluid administered to these patients may be associated with the risk of subclinical kidney injury (16). On the other hand, in older patients limited amounts of intravenous fluids are administered due to the physician’s awareness of age-related pathophysiological changes that predispose older patients to fluid and electrolyte abnormalities and consequently higher morbidity and mortality (17).

## CONCLUSION

In conclusion, it seems that polyuria may act as a predictor of longer duration of hospitalization and cannot predict weaning outcome and is probably associated with a subclinical renal dysfunction. Of course, it is important to emphasize that intravenous fluid should be meticulously administered to critically ill patients to maintain a proper fluid balance.
